# Dementia friends unite! Exploring the effectiveness of a dementia education initiative co‐produced with and for multicultural communities

**DOI:** 10.1002/alz.090138

**Published:** 2025-01-09

**Authors:** Gabriela E Caballero, Emy Tsouros, Mai Tran, Nibras Jasim, Sonia Chan, Angie Dinh, Vivian Dinh, Eliza Chan, Kylie Richardson, Sanna Sartawy, Sharyn Kaapro, Zac Hulm, Joyce Siette, Ann Dadich, Michelle DiGiacomo, Genevieve Z Steiner‐Lim, Diana Karamacoska

**Affiliations:** ^1^ NICM Health Research Institute, Western Sydney University, Sydney, NSW Australia; ^2^ Western Sydney University, Sydney, NSW Australia; ^3^ CASS Care, Sydney, NSW Australia; ^4^ Australian Nursing Home Foundation, Sydney, NSW Australia; ^5^ Myrtle Cottage, Sydney, NSW Australia; ^6^ Dementia Australia Advocates, Sydney, NSW Australia; ^7^ South Western Sydney Dementia Network, Sydney, NSW Australia; ^8^ Harbison Care, Sydney, NSW Australia; ^9^ MARCS Institute for Brain, Behaviour and Development, Western Sydney University, Penrith, NSW Australia; ^10^ University of Technology Sydney, Sydney, NSW Australia; ^11^ Translational Health Research Institute, Western Sydney University, Penrith, NSW Australia

## Abstract

**Background:**

People living with dementia and their carers, particularly those from culturally and linguistically diverse backgrounds, consistently demonstrate limited knowledge about and negative attitudes towards dementia. This can limit access to preventive and post‐diagnostic care. Culturally sensitive dementia education is an inclusive and practical intervention that can be used in multicultural contexts to increase awareness and destigmatise the condition. Our study aimed to explore the reach and effectiveness of a multilingual dementia education initiative, co‐produced by multicultural service providers and people with lived and caring experiences of dementia.

**Method:**

We conducted a quasi‐experimental education intervention targeting English, Arabic, Vietnamese, Cantonese, Mandarin, and Greek‐speaking people in Sydney, Australia. The intervention involved three two‐hour information sessions delivered in‐language by trained bilingual co‐facilitators over three months. The RE‐AIM framework was used to analyse the intervention’s reach and effectiveness. Reach was assessed by the number of attendees at each session. Effectiveness was measured pre‐ and post‐intervention using an adapted survey incorporating 10‐items from the dementia knowledge assessment scale (DKAS) and 14‐items from the dementia attitudes scale (DAS). Mann‐Whitney *U* tests compared mean rank differences pre‐ and post‐intervention for each scale item.

**Results:**

We reached over 220 people across six languages. There were statistically significant improvements from pre‐ to post‐intervention on five DKAS items regarding causes, risk factors, and symptom management (all *p* ≤ 0.050), and eight DAS items regarding perceptions and stigma (all *p* ≤ 0.019).

**Discussion/Conclusion:**

The education initiative was effective in this multicultural context, but ongoing education is needed to address persistent dementia knowledge gaps and negative attitudes. Co‐production with multicultural stakeholders likely enhanced understanding of community needs, resulting in greater information access and acceptability. To measure longer‐term effectiveness, the adapted survey will be re‐administered to capture changes in dementia knowledge and attitudes, 12‐months post‐intervention. While culturally tailored education might overcome nuanced learning and support needs, a culturally sensitive approach is likely to be more effective to enact dementia‐friendly community principles in multicultural contexts.

